# Perspective: Estrogen and the Risk of Cognitive Decline: A Missing Choline(rgic) Link?

**DOI:** 10.1093/advances/nmab145

**Published:** 2021-11-27

**Authors:** Jonathan Bortz, Kevin C Klatt, Taylor C Wallace

**Affiliations:** Balchem Corporation, New Hampton, NY, USA; Baylor College of Medicine, Houston, TX, USA; Think Healthy Group, Washington, DC, USA; Department of Nutrition and Food Studies, George Mason University, Fairfax, VA, USA

**Keywords:** choline, estrogen, cognitive disorders, Alzheimer's disease, brain

## Abstract

Factors that influence the risk of neurocognitive decline and Alzheimer's disease (AD) may provide insight into therapies for both disease treatment and prevention. Although age is the most striking risk factor for AD, it is notable that the prevalence of AD is higher in women, representing two-thirds of cases. To explore potential underlying biological underpinnings of this observation, the intent of this article is to explore the interplay between cognitive aging and sex hormones, the cholinergic system, and novel hypotheses related to the essential nutrient choline. Mechanistic evidence points toward estrogen's neuroprotective effects being strongly dependent on its interactions with the cholinergic system, a modulator of attentional functioning, learning, and memory. Estrogen has been shown to attenuate anticholinergic-induced impairments in verbal memory and normalize patterns of frontal and occipital cortex activation, resulting in a more “young adult” phenotype. However, similar to estrogen replacement's effect in cardiovascular diseases, its putative protective effects may be restricted to early postmenopausal women only, a finding supportive of the “critical window hypothesis.” Estrogen's impact on the cholinergic system may act both locally in the brain but also through peripheral tissues. Estrogen is critical for inducing endogenous choline synthesis via the phosphatidylethanolamine N-methyltransferase (*PEMT*) gene–mediated pathway of phosphatidylcholine (PC) synthesis. *PEMT* is dramatically induced in response to estrogen, producing not only a PC molecule and source of choline for the brain but also a key source of the long-chain ω-3 fatty acid, DHA. Herein, we highlight novel hypotheses related to hormone replacement therapy and nutrient metabolism aimed at directing future preclinical and clinical investigation.

## Teaser

**Statement of Significance**: This perspective identifies numerous areas for future preclinical and clinical research, highlighting the need for randomized controlled trials in postmenopausal women to assess the impact of choline supplementation on cognitive decline, with appropriate consideration of the estrogen availability, critical windows, dose, and PEMT genotype.

## Introduction

As the global population ages, a dramatic increase in age-related neurocognitive decline has been observed. Increasing age is associated with impairments in both brain activity patterns and functional tests of cognition relative to younger adults. Neuroanatomical correlates of this functional decline include increases in brain atrophy (1), particularly in regions of the prefrontal cortex and hippocampus, and diminished localization and coordination of brain activity patterns (2). These age-related declines manifest in susceptible individuals as dementias, the most common being Alzheimer's disease (AD), a progressive neurodegenerative condition that results in initial mild cognitive impairment before progressing to more serious impairment. AD was first described as a rare disorder in 1906 by Alois Alzheimer in the case of a 51-y-old woman; however, modern surveillance data demonstrate that AD accounts for 60–70% of the 50 million cases of dementia worldwide (3). AD is characterized by memory loss, impaired spatial recognition, and difficulty completing the tasks of daily living, impacting the quality of life of not only patients but also their caregivers and placing a significant financial burden on the healthcare system. Thus, it is imperative to identify better interventions to both prevent and treat this disease of aging.

It is well accepted that AD is a complex and multifactorial disease with no single cause; rather, a myriad of genetic and environmental exposures interact to determine risk. Advancing age imparts the greatest risk for developing AD, along with other genetic and lifestyle factors, such as APOE*ε*4 genotype, hypertension, and type 2 diabetes (4). The prevalence of AD is greater in women than men, with current estimates showing that nearly two-thirds of individuals with AD are women (5, 6). While this observation was initially thought to be due to the increased longevity of women, it alone does not appear to explain the discrepancy in susceptibility between men and women; thus, biological variables, such as estrogen, have been the subject of controversy and increased investigation (7).

Maintaining a healthy lifestyle, including adequate nutrition, is a critical component for reducing the risk of cognitive decline during aging and dementia (8). Healthy dietary patterns, such as the Mediterranean dietary pattern, are currently recommended to reduce the risk of cognitive decline for adults with normal or mild cognitive impairments; however, guidance related to optimal intakes of specific dietary components is still an active area of research, with observational studies of nutrient biomarkers demonstrating strong relations to measures of functional brain network efficiency and cognition (9). Unlike pharmacological approaches, nutrition has the potential to exert its effects prior to dementia manifesting or early on in the disease. The essential nutrient choline is one such dietary component that may influence the risk of cognitive decline, as it is the direct precursor to the neurotransmitter acetylcholine, which has been shown to decrease in AD. Less than 3% of women aged ≥71 y in the United States meet the current adequate intake for choline (425 mg/d), a value that is based on prevention of liver dysfunction in young men (10).

In this perspective article, assembled and expanded by the authors after conversations during and after roundtable discussion on February 11, 2020, we explore factors that may contribute to the significantly higher prevalence of AD in women, with an emphasis on the neurocognitive effects of estrogen replacement. Here we also summarize our thoughts on the body of observational and clinical evidence relating estrogen availability to cognitive function and risk of dementia, the role of the cholinergic system in mediating the relation between estrogen availability and cognitive outcomes, and novel considerations for incorporating nutritional neuroscience approaches into this literature base, with a focus on dietary choline intake.

### Estrogen and cognitive function

Interest in the potential neurocognitive effects of estrogen stem from early clinical reports noting changes in cognition, particularly memory, that coincide with female menopause (11, 12). The menopausal transition is associated with increases in follicle-stimulating hormone and a sharp decline in estradiol (13), the latter being strongly associated with hot flashes, the characteristic symptom of this period. The natural menopausal transition occurs in stages of variable lengths, associated with early prolonged cycles followed by intervals of amenorrhea of >60 d (14, 15). Conversely, menopause may occur abruptly, following the surgical removal of the ovaries. Early randomized controlled trial evidence supporting the hypothesis that the endocrine milieu influences cognitive function resulted from examination of the impact of hormonal replacement in surgical menopause and observations of lower cognitive functioning scores (i.e., short- and long-term memory, logical reasoning) in women receiving placebo relative to both women receiving hormonal replacement and patients undergoing hysterectomy but retention of the ovaries (16). Similar results of estrogen are observed when used in “add-back” regimens with gonadotropin-releasing hormone agonists (17). Results of large, modern cohort studies are consistent with those of trials of shorter-term estrogen replacement in surgical menopause and illustrate an increased risk of dementia with oophorectomy (18–20). While such cohort studies are at risk of confounding, causal effects have been argued, owing to key observations (19). First, with regard to dose response, earlier age of surgery is consistently associated with increased risk. Second, surgical indication does not appear to predict disease risk. Finally, subgroups of women undergoing surgery prior to age 49 y and receiving estrogen replacement therapy through the age of 50 show no increased risk of cognitive impairment or dementia (19).

The consistent observational and randomized controlled trial literature of estrogen's impact on cognition following surgical menopause stands in contrast to the more conflicting literature in studies of estrogen and natural menopause. Differences in estrogen's neurocognitive effects may be due to physiological factors, including changes in the endocrine milieu in rapid-onset compared with prolonged menopause, but such inferences are complicated by heterogeneity in study designs. Indeed, studies of natural menopause and estrogen's relation to cognition differ dramatically with respect to study participant chronological age, menopausal status, degree of menopausal symptoms (i.e., hot flashes, impaired sleep), types of hormone replacement therapy utilized, and cognitive domains assessed, challenging interpretation of this literature base.

Observational studies of endogenous estrogen around the time of menopause have revealed conflicting relations with cognitive function. Around the age of the menopausal transition, lower circulating estrogen concentrations have been shown to be directly associated with poorer performance on memory tasks and hippocampal activity and connectivity, as determined by verbal encoding tasks during functional MRI scanning (21). Estrogen's relation to hippocampal activity is consistent with both this brain region's critical involvement in memory and its high expression of estrogen receptors (22). However, other population cohorts, also stratified by menopausal status, have failed to show relations between circulating estrogen and cognitive performance (23, 24). Observational investigations comparing habitual estrogen replacement users relative to nonrecent users have revealed improvements in memory task performance and increased cerebral blood flow, including to the hippocampus (25). Investigations stratifying the effects of estrogen therapy by never-users, perimenopausal users, and postmenopausal users (mean age of 60 y) have shown positive impacts of estrogen replacement on verbal memory and hippocampal activation, exclusive to perimenopausal users (26). The observation that benefits of estrogen replacement may be limited to the perimenopausal window, preventing estrogen decline and its associated cognitive impairments, is referred to as the “critical window hypothesis.” Previous studies in ovariectomized, aged rats demonstrated this effect, showing that the benefits of hormonal therapy on cognitive function and hippocampal function are restricted to a critical window following the loss of ovarian function (27, 28).

Whether these observational studies of estrogen replacement therapy users represent causal effects has been the subject of considerable debate that has spurred numerous short- and long-term randomized controlled trials assessing cognition, risk of dementia, and mortality. While early small trials have found protective effects of estrogen replacement on verbal memory (29–32), recent larger randomized controlled trials have largely found no evidence for protective neurocognitive effects of different estrogen formulations (33–35), including no support for interventions early in the menopausal transition. Notably, formulations including medroxyprogesterone acetate (MPA) in addition to estrogen therapy appear to have a negative impact on verbal memory (36). Null results of estrogen, and detrimental effects of estrogen combined with MPA, have been similarly observed for all-cause dementia (37, 38). In contrast, a significant protective finding of conjugated equine estrogen therapy was observed in the follow-up cohort of the Women's Health Initiative (WHI) randomized trial, demonstrating a 26% reduction in risk of death from AD and other dementia (39). Contrary to the critical window hypothesis, this effect was driven by the subgroup aged 70—79 y. A strength of this WHI cumulative 18-y follow-up study is the relatively large number of subjects and AD cases, long-term follow-up, and ability to assess mortality, whereas other studies with null findings examined dementia incidence in smaller samples over a relatively shorter period (4–5 y), with a substantial percentage of cases being adjudicated (37) (**Table 1**).

**TABLE 1:** Primary RCTs that assess the role of estrogen and cognitive function in postmenopausal women^1^

Reference	Study design	Population	Intervention	Control	Duration	Outcome measure(s)	Outcome assessment(s)	Results
Grady et al. (2002) (89)	RCT (parallel), HERS	Postmenopausal women (age 71 ± 6 y) with coronary disease	0.625 mg/d CEE + 2.5 mg/d MPA (*n* = 517)	Placebo (*n* = 546)	4.2 ± 0.4 y	General cognitive function	MMSE, verbal fluency, Boston Naming, Word List Memory, Word List Recall and Trails B	No observed differences in age-adjusted cognitive test scores except estrogen + progestin scored worse on verbal fluency test (15.9 ± 4.8 vs. 16.6 ± 4.8, *P* = 0.02)
Shumaker et al. (2003) (38)	RCT (parallel), WHIMS	Postmenopausal women free of probable dementia, age 65+ y at baseline	0.625 mg/d CEE + 2.5 mg/d MPA (*n* = 2229)	Placebo (*n* = 2303)	4.05 ± 1.19 y	Incidence of probable dementia and MCI	MMSE	61 women diagnosed with probable dementia, 40 in estrogen + progestin group vs. 21 in placebo group. HR for probable dementia was 2.05 (95% CI, 1.21, 3.48). Treatment effects on MCI did not differ between groups
Shumaker et al. (2004) (37)	RCT (parallel), WHIMS	Postmenopausal women free of probable dementia (age 65–79 y) at baseline	0.625 mg/d CEE-alone or 0.625 mg/d CEE + 2.5 mg/d MPA (*n* = 5157)	Placebo (*n* = 5269)	5.21 ± 1.73 y (CEE-alone) and 4.05 ± 1.19 y (CEE + MPA)	Incidence of probable dementia and MCI	MMSE	Women diagnosed with probable dementia: 28 in estrogen vs. 19 in placebo group. HR for probable dementia: 1.49 (95% CI: 0.83, 2.66); 76 women diagnosed with mild cognitive impairment in estrogen vs. 58 in placebo group. HR for MCI: 1.34 (95% CI: 0.95, 1.89); 178 women diagnosed with probable dementia or mild cognitive impairment in combined estrogen-alone or estrogen + progestin group vs. 132 in placebo group. HR for probable dementia or MCI: 1.41 (95% CI: 1.12, 1.76)
Dumas et al. (2006) (52)	RCT (crossover)	Healthy cognitively normal, older women (age 48–84 y) at baseline	1 mg/d 17-β estradiol (*n* = 15)	Placebo (*n* = 15)	3 mo	Subjects performed cognitive task during 5 anticholinergic challenge sessions and were administered 1 of 2 acute doses scopolamine, mecamylamine, or placebo	Cognitive battery included tests of attention (CFFT, CRT, DSST, DAT, CCPT), verbal learning and memory (BSRT, VPAT, and IPR), and nonverbal learning and memory (RAT and BVRT)	Estrogen pretreatment attenuated anticholinergic drug–induced impairments on tests of attention and tasks with speed components
Resnick et al. (2006) (36)	RCT (parallel), WHIMS	Postmenopausal women free of probable dementia, age 65–79 y at baseline	0.625 mg/d CEE + 2.5 mg/d MPA (*n* = 690)	Placebo (*n* = 726)	Mean 3 y treatment and followed for a mean of 1.35 ± 0.61 y	Annual rates of change in specific cognitive functions (mainly verbal and figural memory), adjusted for time since randomization	WHISCA cognitive battery	Estrogen + progestin had a negative impact on verbal memory (*P* ≤ 0.01) and positive impact on figural memory (*P* = 0.01), compared to placebo. Both effects on memory were evident only after long-term therapy. Other cognitive domains were not significantly affected
Dumas et al. (2008) (53)	RCT (crossover)	Healthy younger (age 50–62 y) and older (age 70–84 y) postmenopausal women at baseline	1 mg/d 17-β estradiol for 1 mo then 2 mg/d for 2 mo (*n* = 22)	1 mg of 17-β estradiol for 1 mo then placebo for 2 mo (*n* = 22)	3 mo	Verbal memory performance and attention after 3 anticholinergic challenge sessions, where subjects were administered an acute dose of scopolamine, mecamylamine, or placebo	Cognitive battery composed of: BSRT, IPR, CFFT, CRT, and CCPT	Estradiol pretreatment significantly attenuated anticholinergic drug-induced impairments on a test of episodic memory (i.e., BSRT) for younger group only, while estradiol treatment impaired performance of older group; study suggests younger subjects may experience greater benefit from estradiol treatment vs. older subjects, supporting concept of a critical period for postmenopausal estrogen use
Gleason et al. (2015) (34)	RCT (parallel), KEEPS–Cog	Recently postmenopausal women (age 52.6 ± 2.6 y)	0.45 mg/d oral CEE + 200 mg/d micronized progesterone (*n* = 220) or 50 μg/d transdermal estradiol + 200 mg/d micronized progesterone (*n* = 211)	Placebo (*n* = 262)	4 y with 2.85 ± 0.49 y follow-up	Verbal learning and memory, auditory attention and working memory, visual attention and executive function, and speeded language and mental flexibility	MMSE	No treatment related benefits found for cognitive outcomes
Henderson et al. (2016) (35)	RCT (parallel)	Healthy women (age 41–84 y) were recruited into early and late postmenopause groups	1 mg/d of 17-β estradiol (*n* = 284)	Placebo (*n* = 283)	57 ± 5.8 mo	Verbal memory	Comprehensive neuropsychological battery (table e-1 on Neurology® website) that emphasized standardized tests sensitive to age-related change	Estradiol did not affect measures of verbal memory, executive functions, or global cognition vs. placebo. Estradiol initiated within 6 y of menopause vs. 10+ y after does not differently affect verbal memory, executive functions, or global cognition
Espeland et al. (2017) (90)	Postintervention follow-up of 2 RCT (parallel) cohorts: WHIMSY and WHIMS-ECO	Younger (age 50–54 y) and older (age 65–79 y) postmenopausal women	0.625 mg/d CEE in women with hysterectomy or 0.625 mg/d CEE + 2.5 mg/d MPA in women without hysterectomy (*n* = 2,103)	Placebo (*n* = 2113)	Follow-up mean 7.2 ± 1.0 y for WHIMSY and 6.4 ± 1.0 y for WHIMS-ECO	Global cognitive functioning, verbal memory, attention, and semantic verbal fluency	Cognitive battery composed of: TICS–m, EBMT, OTMT, and VF–A	Hormone therapy when prescribed to younger women had no significant effect on long-term cognition, but was associated with reduced global cognitive function, working memory, and executive function in older women
Manson et al. (2017) (39)	Postintervention follow-up of 2 RCT (parallel) cohorts: WHI and WHI–OS	Postmenopausal women (age 63.4 ± 7.2 y)	CEE 0.625 mg/d alone or + 2.5 mg MPA (*n* = 13,816)	Placebo (*n* = 13,531)	Cumulative 18-y follow-up	AD or dementia mortality	Regular surveillance of cohort through National Death Index and by reports of next of kin or postal service	Estrogen-alone reduced morality risk from AD or dementia vs. placebo, HR: 0.74 (95% CI: 0.59, 0.94). Estrogen + progestin no effect vs. placebo, HR: 0.93 (95% CI: 0.77, 1.11). Pooled results from 2 cohorts
								suggest hormone therapy may reduce mortality risk from AD or dementia, HR: 0.85 (95% CI: 0.74, 0.98)
Yoon et al. (2018) (91)	RCT (parallel)	Postmenopausal women (age 52–82 y) with multiple-domain, amnestic subtype MCI	0.1%, 2 mg/d percutaneous estradiol gel (*n* = 19)	Placebo (*n* = 18)	24 mo	General cognitive function	ADAS and Korean versions of MMSE and MCA	Dementia progression rates 44.4% (8/18) in estradiol group vs. 52.9% (9/17) in placebo group. Estradiol treatment reduced global cognition deterioration in MCI when adjusted for apoE genotype (ε4 allele) (*P* = 0.0261) and resulted in better cognitive battery scores after 24 mo vs. placebo on both Korean versions of MCA (MD: 3.85; 95% CI: –0.46, 8.16; *P* = 0.043) and MMSE (MD: 3.26; 95% CI: 0.04, 6.48; *P* = 0.0319)
Moradi et al. (2019) (92)	RCT (parallel)	Postmenopausal women (age 55.97 ± 4.92 y) free of chronic disease	0.625 mg/d CEE + 2.5 mg/d MPA + 500 mg/d calcium and 200 IU/d vitamin D tablet (*n* = 70)	500 mg/d calcium and 200 IU/d vitamin D tablet (*n* = 70)	4 mo	General cognitive function and climacteric symptoms	MCA and GCS	All MCA domains except orientation (i.e., visuospatial/executive, memory, attention, language, abstraction, total MCA score GCS improved in estrogen and progestin group vs. control group (*P* < 0.001) MCA and GCS negatively correlated after intervention (*r* = –0.235; *P* = 0.006)

^1^Values presented as means ± SDs unless otherwise indicated. AD, Alzheimer's disease; ADAS, Alzheimer's Disease Assessment Scale; BSRT, Buschke Selective Reminding Task; BVRT, Benton Visual Retention Test; CEE, Conjugated Equine Estrogens; CFFT, Critical Flicker Fusion Task; CCPT, Connors Continuous Performance Test; CRT, Choice Reaction Time task from the Milford Test Battery; DDST, Digit Symbol Substitution Test; DAT, Divided Attention Test; EBMT, East Boston Memory Test; GCS, Green Climacteric Scale; HERS, Heart and Estrogen/Progestin Replacement Study; IPR, New York University (NYU) Immediate Paragraph Recall; KEEPS–Cog, Kronos Early Estrogen Prevention Study–Cognitive Affective Study; MCA, Montreal Cognitive Assessment; MCI, mild cognitive impairment; MMSE, Mini-Mental State Examination; MPA, medroxyprogesterone acetate; OTMT, Oral Trail Making Test; RAT, Repeated Acquisition Task; TICS–m, Telephone Interview for Cognitive Status–modified; VF–A, Verbal Fluency–Animals; VPAT, Verbal Paired Associates Test; WHI, Women's Health Initiative; WHIMS, Women's Health Initiative Memory Study; WHIMS–ECHO, Women's Health Initiative Memory Study–Epidemiology of Cognitive Health Outcomes; WHIMSY, Women's Health Initiative Memory Study of Younger Women; WHI–OS, Women's Health Initiative–Observational Study.

Although the literature base to inform the therapeutic use of estrogen replacement to decrease cognitive decline is mixed, there may be future avenues for research given the general trend toward a benefit of estrogen, particularly from animal models, studies of surgical menopause, and in those investigations with long-term follow-up. There is a need for more targeted trials to identify individuals most likely to benefit from estrogen therapy, with such added precision informed by a better mechanistic understanding of estrogen's effects.

### Effect mediators and modifiers of the estrogen–cognition link

To date, much of the existing scientific literature has excluded women reporting symptoms of hot flashes. Objectively measured hot flashes are associated with temporarily impaired verbal memory, independent of menopausal status (40). Cortisol spikes associated with hot flashes (41) likely mediate the detrimental relation between hot flashes and acute cognitive impairments. Importantly, estrogen appears to mitigate the detrimental effects of cortisol on cognitive function (42), suggesting that the cognitive vulnerability associated with hot flashes may indeed open a therapeutic role for estrogen. Thus, intervention trials excluding individuals experiencing hot flashes also excluded individuals with substantial potential to benefit from these intervention trials.

### Estrogen–neuronal cholinergic system interactions

Heterogeneity in the literature base surrounding estrogen's impact on cognitive function indicates that a better understanding of relevant mechanisms at play may provide clarity on both the direct effect of estrogen and the possible biological underpinnings of suggested moderating factors (e.g., the critical window hypothesis and its correlates). The cholinergic system is well recognized as being critical for cognitive function, including attention, working memory, information filtering, and performing effortful tasks. Numerous age-associated defects in the cholinergic system have been characterized and this information has led to the development of the cholinergic hypothesis of cognitive aging and AD (43). The hypothesis originated from observations that, in AD, acetylcholine synthesis and cholinergic receptor signaling is disturbed in numerous brain regions, including the cortex. Indeed, acetylcholinesterase inhibitors remain a mainstay for treatment of mild cognitive impairment and early AD (43).

This critical role of the cholinergic system in cognition throughout the lifespan has led researchers to hypothesize that estrogen's impact on cognition is dependent on intact cholinergic signaling. Early evidence to support this hypothesis is derived from the observation that ovariectomized rats exhibit decreased choline uptake, as well as decreased choline acetyltransferase mRNA and activity in the hippocampus, while exhibiting impaired performance of learning and memory tasks (44–46). Notably, replacement of estrogen in this context is able to recover choline acetyltransferase activity levels and improve cognition following ovariectomy (47). However, cognitive improvements are not observed when estrogen is given following cholinergic system lesioning by the selective cholinergic immunotoxin 192 IgG-saporin (48) or scopolamine (49), an antagonist to the muscarinic receptor family of the cholinergic system. Hippocampal M2 muscarinic receptors appear particularly critical for mediating estrogen-induced improvements in working memory (50). Findings of estrogen-induced improvements in cognitive function that require intact cholinergic signaling are apparent not only in rodents but also in primate models of surgical menopause (51), suggesting an evolutionary conserved axis involving sex steroids and hippocampal cholinergic signaling to modify cognitive processes.

Translating these preclinical findings to humans has relied on pharmacological approaches to block cholinergic signaling through nicotinic (i.e., with mecamylamine) and muscarinic (i.e., with scopolamine) receptors. Early clinical investigations demonstrated that 1 mg/d of oral estradiol for 3 mo was able to largely blunt the impairments in attention and speed from either receptor antagonist in 15 postmenopausal women, although effects were less pronounced for verbal episodic memory (52). Future investigations by this laboratory group furthered these findings by providing 1 mg/d oral estradiol for 1 mo, followed by 2 mg/d oral estradiol for 2 mo across 2 groups of healthy younger (aged 50–60 y) and older (70–81 y) postmenopausal women (53). The effects of estradiol treatment on measures of episodic verbal memory (i.e., total recall, recall consistency, and recall failure) following scopolamine challenge were dependent on age, with estrogen significantly attenuating the effects of muscarinic antagonism in younger postmenopausal women but not in the older group. Notably, older postmenopausal women receiving estradiol performed nominally worse than those who received a placebo. These results collectively suggest that the apparently conflicting literature base on estrogen replacement in menopause and cognition outcomes may result from interactions between both menopausal state and the degree of cholinergic impairment present. This observation provides a rationale for additional pharmacological functional neuroimaging studies, including the need to consider estrogen replacement's interactions with not only antagonists but also enhancing compounds (e.g., acetylcholineesterase inhibitors), and to identify readily measurable correlates of intact or disrupted cholinergic signaling that can be readily measured and used in the inclusion/exclusion criteria of estrogen replacement trials.

### Estrogen, choline, and brain aging

Estrogen's link to the cholinergic system is not exclusive to its impact in neurons; indeed, it has been appreciated for decades that female sex hormones play a protective role against dietary choline deficiency in mammals (54), including humans (55). Choline, similar to vitamin D, can be endogenously synthesized, thus influencing the requirement from diet. The liver is capable of producing a choline moiety de novo from the triple methylation of phosphatidylethanolamine to phosphatidylcholine (PC) via the action of the phosphatidylethanolamine N-methyltransferase (*PEMT*) gene (55, 56). The promoter region of *PEMT* contains an estrogen response element (ERE) and its mRNA and activity are dramatically upregulated in response to estrogen exposure (57), buffering premenopausal women and postmenopausal women taking estrogen replacement therapy from the organ dysfunction associated with consuming a choline-deficient diet (55, 58). The PC produced by the *PEMT* pathway can be hydrolyzed by either phospholipase D to yield a free choline moiety or by phospholipases to produce a lysophosphatidylcholine, both readily crossing the blood–brain barrier (59, 60). Notably, *PEMT* not only provides a source of choline but also produces a PC species enriched with the ω-3 fatty acid DHA (61, 62), well recognized for its role in maintaining cognitive function. PC and DHA are critical for maintaining membrane integrity that may independently influence cognition; indeed, higher blood concentrations of PC (63) and DHA (64, 65) are beneficially associated with diverse measures of cognitive function. Both PC and DHA also serve to support and enhance cholinergic signaling, with PC serving as a repository of choline for acetylcholine synthesis (66) and DHA facilitating cholinergic transmission (67). Thus, *PEMT* serves as an estrogen-inducible regulator of the supply of choline to the brain, the rate-limiting step in acetylcholine synthesis (66, 68), and further provides DHA to facilitate cholinergic signaling.

To date, studies of estrogen replacement, cognition, and dementia risk have not considered choline supply, from endogenous synthesis or exogenously from diet, as a key moderating factor. Measuring choline flux throughout body compartments, including the brain, is challenging due to both practical and safety concerns. However, natural experiments may provide the unique opportunity to test the impact of estrogen on cognition and the dependence of choline supply. Common single nucleotide polymorphisms (SNPs) exist in linkage disequilibrium in the *PEMT* gene, including the functionally characterized SNP, rs12325817 G→C; this SNP exists within the promoter region of *PEMT*, proximal to the ERE, and abrogates estrogen-induced increases in *PEMT* gene expression (69). When women, both premenopausal and postmenopausal, are given choline-deficient diets for consumption, individuals harboring this variant exhibit increased risk of organ dysfunction (58, 70), presumably by influencing the capacity to upregulate endogenous choline production. Notably, 24% of European American women are homozygous for the *PEMT* rs12325817 effect allele and thus require additional dietary choline to meet tissue choline needs (71). Similar to assessments of the interactions of estrogen with pharmacological inhibitors of cholinergic signaling, studies assessing estrogen's impact on cognition in individuals harboring common variants in the *PEMT* gene that abrogate estrogen-induced binding can be performed. Such physiological studies may be combined with post hoc analyses of large existing estrogen replacement trials to assess estrogen's -interaction with *PEMT* genotype to inform future prospective studies.

The degree to which *PEMT* genotype compromises choline supply depends, in part, on dietary choline intakes. At present, there is substantial uncertainty regarding dietary choline needs throughout the life course. Current dietary recommendations for adults are derived from a single study in men that monitored markers of liver and muscle dysfunction (72, 73). Only scarce data in humans inform the dose–response relation between choline intake and indicators of function of other organs, such as the brain. This scarcity of information contrasts with animal models with which decades of investigations have demonstrated that perinatal and lifelong dietary choline intakes that are higher than those required to meet basal requirements for preventing hepatic dysfunction influence cognition across numerous animal models (74). Typically, choline intakes 4 to 5 times those of usual chow intakes are utilized, suggesting that significantly higher intakes of choline than are required to meet basal needs result in improved cognition. Although the findings are difficult to translate to humans, observations of rodent models have revealed beneficial effects of choline supplementation in reducing AD-like histological and cognitive abnormalities when provided across the lifespan (75), during adulthood (76), and at the time of gestation (77), with the latter demonstrating intergenerational reductions in plaque numbers in offspring born to choline-supplemented dams despite normal choline intakes in the offspring. Unfortunately, no investigations in rodent models have carefully analyzed the cognitive impacts of high-choline diets following ovariectomy. Although choline has broad pleiotropic effects, including serving as a major dietary methyl donor, few studies have specifically examined choline supplementation's interactions with genetic or pharmacological disruptions to highlight a specific mechanism of action. Enhanced cholinergic action, epigenetic modifications, and reduced homocysteine have all been put forth to explain the observed cognitive benefits of choline supplementation (74). Although some evidence demonstrates loss of betaine hydroxymethyl transferase, responsible for the irreversible oxidation of choline to betaine and its role as a 1-carbon donor, results in reduced total brain volume and impaired reference memory (78), these results are challenging to disentangle from the concomitant rise in homocysteine. Dietary choline's interaction with cholinergic signaling is likely to be a primary mediator of its neuroprotective effects, given the potent anti-inflammatory roles of the α7 nicotinic receptor in dampening down the microglia- and astrocyte-mediated neuroinflammation commonly observed in neurocognitive disease (79, 80). Despite an elusive mechanism, such studies underlie the enthusiasm for enhancing choline supply through exogenous provision of choline in the diet to beneficially impact cognition.

Choline is already included as an ingredient in a medical food intended for the treatment of AD (81). Several human studies support the hypothesis that higher choline intakes may be associated with a lower risk of incident dementia. A recent prospective analysis of the Kuopio Ischemic Heart Disease Risk Factor Study showed dietary choline intake to be inversely associated with risk of incident dementia and cognitive performance in middle-aged to older men (82). Clinical studies supporting a relation between choline intakes and cognitive function include reports of choline-responsive verbal and visual memory impairments in individuals receiving total parenteral nutrition (83) as well as higher self-reported choline intakes being modestly associated with cognitive function and little to no white matter volume in mid- to late life (84). A multicenter randomized intervention of patients affected by mild to moderate AD showed improvements in all assessed cognitive parameters in those treated with 400 mg choline alfoscerate, a semisynthetic derivative of phosphatidylcholine, 3 times/d for 180 d (*n* = 132; 105 females) compared with placebo (*n* = 129; 94 women) (85). Additionally, a small body of highly heterogeneous intervention trials exist that report mixed and inconsistent effects of choline supplementation on select tests of cognitive domains in adults (86). More promising data exist for the impact of maternal choline supplementation on fetal development and infant cognition (87, 88). While these findings suggest that dietary choline intakes can influence cognition, this body of evidence is limited by a lack of consideration for sex and menopausal status interactions as well as poorly characterized dose–response relations. Thus, controlled feeding and supplementation trials of choline across a range of doses, carefully accounting for sex, menopausal status and timing, and *PEMT* genotype, in addition to well-known risk factors (e.g., ApoE genotype), are needed to assess choline's potential role in promoting neurocognition, particularly for enhancing the effects of estrogen replacement.

## Conclusions

The role of estrogen replacement therapy in reducing the risk of cognitive decline as well as dementia-related morbidity and mortality remains an active area of research. Although the literature is highly heterogeneous and presents mixed results, signals for benefit exist and future research is needed to identify factors that may influence the response to replacement. Indeed, the importance of effect modification has been key to advancing this field of investigation forward, with early emphasis on menopausal timing as a key factor. This perspective presents a testable working model of estrogen's relation to cognition with novel effect modifiers, including the degree of cholinergic dysfunction and the availability of choline from endogenous and exogenous sources. This perspective also shows novel routes of investigation related to estrogen replacement and its interactions with menopausal timing, cholinergic signaling, and the influence of the endogenous (i.e., common *PEMT* variants) and exogenous (i.e., dietary) choline supply on cognitive function and risk of age-related cognitive decline and dementia.

## Notes

Funded by the Balchem Corporation.

Author disclosures: JB is the Vice President of Nutrition Science for Balchem Corporation. KCK and TCW have received research funding from Balchem Corporation. TCW has received additional research funding from the Egg Nutrition Center and National Cattlemen's Beef Association for various research endeavors relating to choline.

Perspective articles allow authors to take a position on a topic of current major importance or controversy in the field of nutrition. As such, these articles could include statements based on author opinions or point of view. Opinions expressed in Perspective articles are those of the author and are not attributable to the funder(s) or the sponsor(s) or the publisher, Editor, or Editorial Board of Advances in Nutrition. Individuals with different positions on the topic of a Perspective are invited to submit their comments in the form of a Perspectives article or in a Letter to the Editor.

Abbreviations used: AD, Alzheimer's disease; ERE, estrogen response element; MPA, medroxyprogesterone acetate; PC, phosphatidylcholine; *PEMT*, phosphatidylethanolamine N-methyltransferase; SNP, single nucleotide polymorphism; WHI, Women's Health Initiative
